# Optical coherence tomography is a useful tool in the differentiation between true edema and pseudoedema of the optic disc

**DOI:** 10.1371/journal.pone.0208145

**Published:** 2018-11-29

**Authors:** Arturo Carta, Paolo Mora, Raffaella Aldigeri, Fabrizio Gozzi, Stefania Favilla, Salvatore Tedesco, Giacomo Calzetti, Roberta Farci, Piero Barboni, Stefania Bianchi-Marzoli, Maurizio Fossarello, Stefano Gandolfi, Alfredo A. Sadun

**Affiliations:** 1 Ophthalmology Unit, Department of Medicine and Surgery, University of Parma, Parma, Italy; 2 Department of Medicine and Surgery, University of Parma, Parma, Italy; 3 Independent Researcher, Parma, Italy; 4 Ophthalmology Unit, University Hospital of Cagliari, Cagliari, Italy; 5 “Studio Oculistico d’Azeglio”, Bologna, Italy; 6 Instituto Auxologico Italiano IRCCS, Neuro-Ophthalmology Center, Scientific Institute Capitanio Hospital, Milano, Italy; 7 Doheny Eye Institute, Department of Ophthalmology, David Geffen School of Medicine, UCLA, Los Angeles, California, United States of America; Bascom Palmer Eye Institute, UNITED STATES

## Abstract

**Purpose:**

To assess the usefulness of spectral-domain optical coherence tomography (SD-OCT) peripapillary retinal nerve fiber layer (RNFL) thickness measurement in discriminating early phase optic disc edema (ODE) from pseudoedema (PODE).

**Methods:**

Hospital-based, multicenter, cross-sectional study involving external patients referred for recent identification of “presumed ODE”. Patients underwent SD-OCT optic nerve head (ONH) RNFL thickness measurement at their first evaluation. In 155 of these, the causative etiology was subsequently ascertained and the respective eyes (one per patient) were assigned to the ODE (95 eyes) or PODE (60 eyes) group. Admission SD-OCT data were retrieved and used for the analysis. ROC curve analysis was used to calculate specificity, sensitivity and predictive value (PV) of the RNFL values.

**Results:**

The PODE group was significantly younger than the ODE group (p = 0.007). The average and any single-quadrant RNFL thickness was significantly higher in the ODE group compared with the PODE and control groups. The average and the inferior quadrant thicknesses tested the most powerful parameters to differentiate ODE from PODE. A cutoff value of ≥ 110 μm for the average area, or of ≥ 150 μm for the inferior quadrant was associated with maximal sensitivity and specificity with positive PV greater than 80%.

**Conclusions:**

The SD-OCT evaluation of the peripapillary RNFL achieved good specificity, sensitivity and positive PV in discriminating between ODE and PODE. Despite the correct differential diagnosis between these categories still relies on a careful medical history taking and other ancillary testing, we proved the usefulness of SD-OCT RNFL measurement in supporting the diagnostic process.

## Introduction

Optic disc edema (ODE) encompasses various findings related to the elevation of the optic nerve head (ONH) in relation to the retinal plane. The presence of mono- or bilateral edema of the optic disc usually represents a very serious evidence because of its potential as an indicator of severe neuro-ophthalmological disease, that may be visual and, at times, life-threatening. A prompt distinction between true ODE and certain variants of the ONH morphology mimicking, what is commonly called pseudoedema (PODE), is crucially important. In the event of ODE, there may be urgency in management as the prognosis often depends on the latency to proper treatment. With PODE, instead, some invasive and potentially harmful tests could be avoided or postponed, as it usually runs a more benign course. Considering current first-line examinations, the effectiveness of funduscopy is markedly operator-dependent and there is no objective metric for indexing severity. This may explain the high misdiagnosis percentage (about 40% in adults, up to 76% in children at their first fundus examination), where misdiagnosis usually occurs when funduscopy is not performed by a skilled physician [1–3]. In fluorescein angiography (FA) a late staining of the optic disc is a highly specific finding of optic disc drusen; a progressive leakage is pathognomonic of true ODE [3,4]. FA, however, is somewhat invasive and not always viable in pediatric patients.

The currently available optical coherence tomography technology [i.e. spectral domain optical coherence tomography (SD-OCT)] enables extremely fast, noninvasive scanning of the ONH structure with an excellent repeatability that is marginally operator-dependent. SD-OCT has been a consistent way to detect disorders involving thinning of the peripapillary retinal nerve fiber layer (RNFL) as a major finding, which commonly occurs in glaucoma. Depletion of the RNFL is currently assessed by SD-OCT even in patients affected by other chronic neurodegenerative disorders, such as Alzheimer’s disease, Parkinson’s disease, and multiple sclerosis [5–7]. Little less is known about the reliability of SD-OCT measurements of cases in which RNFL swelling is the dominant pathological feature [2, 8–10].

Present evidences are generally in agreement on the suitability of SD-OCT in identifying and characterizing patients with abnormally elevated optic discs; nonetheless they found that the peripapillary RNFL thickness alone is hardly enough for defining the specific etiology of acute ONH edema [11–15]. Furthermore, OCT evaluation of the optic nerve head is considered a second level test when approaching patients presenting with an elevated optic disc. The present study was aimed to investigate the usefulness of SD-OCT RNFL thickness measurements in supporting the early process of distinction between ODE in the acute phase (any form) and other clinical entities (for example, bilateral optic disc drusen) which could resemble edema but are actually PODE. Such an early differentiation could valuably assist the appropriate and correct scheduling of further tests (some of which may be very invasive and expensive) required to properly manage each specific case.

## Materials and methods

This was a multicenter, hospital-based, cross-sectional study evaluating a diagnostic procedure for patients with presumed ODE, performed from September 2014 until August 2017. The study was approved by the Institutional Review Board of the University of Parma and it adhered to the tenets of the Declaration of Helsinki. All the participants (no minors were included) gave their verbal informed consent to analyze data obtained at their baseline evaluation. Outpatients referred from physicians to the involved neuro-ophthalmological Services for “presumed ODE” in the acute phase (i.e., reported for the first time within the last two weeks) were deemed eligible and underwent SD-OCT RNFL thickness measurement at the admission. Exclusion criteria for the succeeding enrollment were the following: any previous history of chronic/degenerative ocular or cerebral disease; eyes with high hyperopia or myopia (> 6.00 diopters); low quality SD-OCT images related to improper disc centering, or a signal strength < 5. All eligible subjects underwent a complete neuro-ophthalmological evaluation consisting of: detailed general and ocular history (with particular attention to the ocular major complaints and possible related extraocular symptoms); natural and best-corrected distance and near visual acuity; pupillary reflex testing; intraocular pressure measurement; Ishihara’s color plate testing; slit-lamp biomicroscopy; fundus evaluation under mydriasis by indirect ophthalmoscopy and fundus color photography; automated visual field testing. After this general assessment, in order to ascertain the causative etiology responsible for the elevation of the optic disc, the diagnostic work-up became case-specific and, for most of the subjects, multidisciplinary. These supplemental investigations were as follows: serology for autoimmunity markers (in 59 subjects), and antibody levels for infectious diseases (in 43 subjects); autofluorescence and FA (in 114 subjects); B-scan ultrasound (in 31 subjects); visual evoked potentials (in 23 subjects); cerebral computed tomography (CT) scan (in 78 subjects); cerebral magnetic resonance imaging (in 67 subjects); magnetic resonance angiography (in 11 subjects); lumbar puncture (in 35 subjects); mitochondrial DNA analysis (in 15 subjects); surgical biopsy (in 8 subjects). The clinical course (after at least 3 months of follow up) of each patient had to be compatible with the designated etiology to make the subject finally labelled as ODE or PODE.

Among all SD-OCT data collected at the admission visit, the study considered only those from patients with a definitive diagnosis, achieved via the above mentioned post-admission work-up. In cases showing bilateral simultaneous involvement, the right eye was included in the study. An additional group of 208 control eyes was created by performing a similar SD-OCT RNFL thickness measurement in willing patients consulted for possible refractive anomalies, strabismus, or choroidal nevi. These patients were checked for acceptable refraction, intraocular pressure within normal limits, normal biomicroscopy, and regular ONH (as detected by funduscopy); only one eye of each control was selected for the purpose of our statistical analysis.

All subjects underwent RNFL thickness measurements using the Cirrus HD-OCT 4000 (Carl Zeiss Meditec, Dublin, CA, USA). This instrument has proven high reproducibility for average, quadrant, and clock-hour RNFL thickness measurements [16–18]. A skilled optometrist blinded to the clinical status of the participants performed the image acquisition using the Optic Disc Cube 200 × 200 protocol. Before accepting the values, one of the authors (A.C.) confirmed the quality of each scan and the correct identification of the limits of the RNFL by the OCT software. The average RNFL thickness, and those of the four principal quadrants (i.e., inferior, superior, nasal, and temporal), were analyzed. Because of the well-established physiological decrease in RNFL thickness with age, statistical evaluations also considered for three age groups: ≤ 35 years, 36–63 years, and ≥ 64 years [19].

Categorical data are presented as absolute numbers (percentages); continuous data are presented as means ± standard deviation (SD), or as median values and interquartile ranges (25–75%).

To assess the variability of SD-OCT parameters, the Coefficient of Variation was calculated. The normality of the distribution of the data was tested using the Kolmogorov-Smirnov test. A bootstrap method for matched pairs, stratified according to sex and age, was used to obtained samples from the control group (the largest), to enable a balanced and homogeneous comparison with the SD-OCT data of the ODE and PODE groups. For each pair of groups, a statistical comparison was performed using the Kruskal-Wallis test, Mann-Whitney U test, or chi-square test, as appropriate. Correlations were tested using Spearman’s rho test. All analyses were two-tailed with significance set at p < 0.05.

For the OCT parameters, a receiver operating characteristic (ROC) analysis was performed and the area under the curve (AUC) was estimated. The Youden’s index (YI) and diagnostic odds ratio were used as diagnostic accuracy indexes to identify cutoff values. The sensitivity, specificity, accuracy (as proportion correctly classified), proportion incorrectly classified (PIC), positive predictive value (PPV), and negative predictive value (NPV) were determined for the identified cutoff values. Statistical analysis was performed using SPSS for Windows statistical software (ver. 24.0; SPSS Inc., Chicago, IL, USA).

## Results

Of the 202 patients consecutively referred for presumed ODE, 191 were eligible for inclusion in this study. A specific etiological diagnosis was obtained for 184 patients, and the respective SD-OCT scan at admission was qualitatively acceptable in 155 patients (73 males and 82 females; mean age, 45 ± 21 years, all of Caucasian ethnicity). Only one eye of each of these 155 patients was selected for the study following the criteria described in the Patients and Methods section. The ODE group consisted of 95 eyes [corresponding to 95 patients; mean age, 50 ± 21 years; sex (M/F), 47/48]. The diagnoses involved 43 eyes with papillitis, 18 eyes with non-arteritic anterior ischemic optic neuropathy (NAION), 16 eyes with arteritic anterior ischemic optic neuropathy (AAION), 13 eyes from patients with idiopathic intracranial hypertension (IIH), and 5 eyes with central retinal artery occlusion. The PODE group consisted of 60 eyes [corresponding to 60 patients; mean age, 41 ± 21 years; sex (M/F), 26/34] involving 28 eyes with physiological variants of optic disc morphology (8 eyes with “crowded” and 20 eyes with “tilted” optic discs), 11 eyes with optic disc drusen (mainly deep drusen), 8 eyes with myelinated fibers, and 13 eyes of asymptomatic carriers of the mtDNA mutation for Leber’s Hereditary Optic Neuropathy (LHON) referred since they were relatives of full-blown patients [20,21].

No statistical difference was found between the two study groups in terms of gender or laterality. The PODE group was significantly younger than the ODE group (p = 0.007). SD-OCT parameters of the ODE, PODE, and control groups, and significant p-values for pair comparisons, are listed in Table 1.

**Table 1:** OCT data in the study groups and controls.

		A-RNFL	T-RNFL	S-RNFL	N-RNFL	I-RNFL
**ODEn = 95**	Median thickness(µm)[IQR]	141[102,218]	89[63, 144]	167[115,273]	101[71,174]	193[132–306]
	CV	53%	70%	60%	69%	53%
**PODEn = 60**	Median thickness (µm) [IQR]	96[84,110]	69[59, 79]	117[92,141]	72[62,88]	127[103–146]
	CV	25%	32%	36%	27%	29%
**Controlsn = 208**	Median thickness (µm)[IQR]	94[88,100]	64[58,71]	117[107,127]	71[64,79]	121[113–134]
	CV	11%	15%	13%	17%	14%
	**significant p-value**	**<0.0001** * †	**<0.0001** * †	**<0.0001** * †	**<0.0001** * †	**<0.0001** * †

RNFL = retinal nerve fiber layer; CV = coefficient of variation; ODE = optic disc edema; PODE = pseudoedema; A-RNFL = average RNFL; T-RNFL = temporal RNFL; S-RNFL = superior RNFL; N-RNFL = nasal RNFL; I-RNFL = inferior RNFL. The p-value refers to the comparison between:

* ODE vs. Controls,

^†^ ODE vs. PODE.

Average and any single-quadrant RNFL thickness was significantly higher in the ODE group compared with both the PODE and control groups. No statistical difference was found between the PODE group and the control group in regards to RNFL. RNFL thickness in the control group showed the lowest Coefficient of Variation. PODE eyes showed a significant negative correlation (p<0.001) between age and RNFL thickness, especially in the superior (r = -0.352), inferior (r = -0.294), and average (r = -0.280) areas. A similar negative correlation characterized the control group, involving the superior (r = -0.261), average (r = -0.248), and temporal (r = -0.200) quadrants. There was no correlation between age and any RNFL thickness measurement in the ODE group.

To identify the most suitable cutoff value to discriminate between the ODE and PODE groups, ROC analyses for RNFL thickness were performed. The curves were calculated by referring to either the overall groups (i.e. without age subdivision), or to age tertiles. Overall analyses showed that average and inferior areas achieved the highest accuracy, with an AUC of 0.785 [standard error (SE) = 0.037] and 0.777 (SE = 0.037), respectively (Table 2). This high discriminative capacity of the average and inferior areas was confirmed using split-age analysis, the age tertile of ≤ 35 years having the lowest AUC.

**Table 2:** Area under the curve (AUC) for ODE versus PODE in overall groups and in age-split analyses.

		Overall	≤35 yrs	36–63 yrs	≥64 yrs
Average RNFL	AUC	0.785	0.721	0.846	0.839
	SE	0.037	0.073	0.053	0.059
	p-value	<0.001	0.007	<0.001	<0.001
Inferior RNFL	AUC	0.777	0.738	0.792	0.883
	SE	0.037	0.071	0.062	0.050
	p-value	<0.001	0.004	<0.001	<0.001
Superior RNFL	AUC	0.736	0.708	0.764	0.801
	SE	0.040	0.074	0.069	0.070
	p-value	<0.001	0.011	0.005	0.002
Nasal RNFL	AUC	0.723	0.651	0.724	0.785
	SE	0.041	0.081	0.070	0.069
	p-value	<0.001	0.064	0.007	0.004
Temporal RNFL	AUC	0.684	0.718	0.650	0.691
	SE	0.043	0.72	0.076	0.077
	p-value	<0.001	0.008	0.069	0.053

AUC: Area under the curve; SE: standard error; RNFL: retinal nerve fiber layer thickness

In a similar manner, the ROC curves comparing ODE and matched samples of control eyes confirmed the high discriminative capacity of the average and inferior quadrant thicknesses [AUC = 0.832 (95%CI 0.769–0.895) and AUC = 0.809 (95%CI 0.741–0.877), respectively in the whole sample]; the youngest tertile showed again the lowest AUC (Table 3).

**Table 3:** Area under the curve (AUC) for ODE versus CONTROLS in overall groups and in age-split analyses.

		Overall	≤35 yrs	36–63 yrs	≥64 yrs
Average RNFL	AUC	0.832	0.791	0.877	0.821
	SE	0.032	0.067	0.045	0.059
	p-value	<0.001	<0.001	<0.001	<0.001
Inferior RNFL	AUC	0.809	0.776	0.825	0.815
	SE	0.035	0.071	0.055	0.059
	p-value	<0.001	<0.001	<0.001	<0.001
Superior RNFL	AUC	0.754	0.777	0.675	0.822
	SE	0.038	0.069	0.071	0.061
	p-value	<0.001	<0.001	0.015	<0.001
Nasal RNFL	AUC	0.748	0.672	0.740	0.797
	SE	0.039	0.080	0.066	0.062
	p-value	<0.001	0.022	0.001	<0.001
Temporal RNFL	AUC	0.740	0.747	0.759	0.716
	SE	0.038	0.068	0.065	0.073
	p-value	<0.001	0.001	<0.001	0.002

AUC: Area under the curve; SE: standard error; RNFL: retinal nerve fiber layer thickness.

Based on these results, the average and inferior thicknesses were chosen as reference parameters to differentiate the ODE and PODE groups. The highest value of YI (0.46) in the overall analysis guided the selection of the most suitable discriminating cutoffs (Table 4).

**Table 4:** ODE versus PODE; SE, SP, accuracy, PIC, PPV, NPV for chosen cutoff values of RNFL thickness (average and inferior) in overall and age-split analyses.

		Overall	≤35 yrs	36–63 yrs	≥64 yrs
Average RNFL ≥ 110 µm	SE	0.70	0.64	0.71	0.72
	SP	0.76	0.60	0.83	0.92
	Accuracy	0.72	0.60	0.76	0.72
	PIC	0.28	0.40	0.24	0.28
	PPV	0.82	0.64	0.86	0.96
	NPV	0.61	0.60	0.66	0.55
Inferior RNFL ≥ 150 µm	SE	0.67	0.71	0.70	0.63
	SP	0.80	0.68	0.77	1.00
	Accuracy	0.70	0.70	0.69	0.72
	PIC	0.28	0.30	0.31	0.28
	PPV	0.83	0.71	0.82	1.00
	NPV	0.61	0.68	0.63	0.5
Average RNFL≥ 110 µmorInferior RNFL ≥ 150 µm	SE	0.74	0.75	0.74	0.72
	SP	0.72	0.70	0.74	0.92
	Accuracy	0.73	0.68	0.74	0.77
	PIC	0.27	0.32	0.26	0.23
	PPV	0.81	0.68	0.81	0.96
	NPV	0.63	0.68	0.65	0.55

SE: sensitivity, SP: specificity, PIC: proportion incorrectly classified, PPV: Positive predictive value, NPV: Negative predictive value.

Concerning the average area, a thickness ≥ 110 μm was associated with maximal sensitivity and specificity (0.70 and 0.77, respectively) and PPV value of 0.82. The proportion correctly classified or accuracy reached 72%. Regarding the inferior quadrant, thicknesses ≥ 150 μm corresponded to a sensitivity of 67%, specificity of 80%, with a PPV of 83%. The presence of average thickness ≥ 110 μm or thickness of the inferior quadrant ≥ 150 μm increased both sensitivity and accuracy. These thresholds were maintained or improved when calculated in the intermediate and oldest age tertiles.

## Discussion

The differential diagnosis of ODE from PODE is common and important, yet remains a significant clinical challenge. The present study investigated the usefulness of SD-OCT RNFL measurements in supporting an early selection of those cases who are more likely true ODE, and with an indication for prompt and extensive management. Different causes of ODE and PODE were merged in our analyses; this was done because it closely resembles the daily clinical practice in a hospital referral center, and because the distinction between every different etiology of ODE or PODE would have provided us with classes too small for a robust statistical evaluation. Based on the data analysis, we conclude the following. 1) The PODE group was significantly younger than the ODE group and 2) PODE eyes showed a significant negative correlation between age and RNFL thickness. Possible explanations for these demographic findings encompass the congenital basis of most PODE instead of the acquired origin of most ODE. Moreover, the age-related RNFL thinning in PODE eyes could reduce the likelihood for a PODE first observed in an elder age to be considered as having an alarming ONH aspect. 3) In our series the most powerful RNFL parameters to discriminate ODE eyes from PODE were the average and inferior quadrant thicknesses. This was true for the groups as a whole, and in the age group sub-analyses, with more significant p values in the elder age tertiles. The above-mentioned age-related RNFL thinning in PODE cases might have influenced this latter evidence. To explain the discriminatory ability of quadrants on the vertical axis of the ONH (chiefly the inferior quadrant) we made the following assumption; because the swelling of a true ODE is mostly due to blocked axoplasmic transport and subsequent intracellular accumulation of organelles, swelling will be more pronounced in large caliber axons originating from large retinal ganglion cells which typically are located at the inferior and superior poles of the ONH [22,23]. By contrast, axoplasmic flow is not blocked in PODE. Previous studies showed variable results in this respect, but rather identifying the nasal quadrant as the most important for differentiating ODE from PODE [2,8,24]. Most of these studies, however, focused on differentiating ODE from optic disc drusen, which typically locate nasally thus causing displacement and thinning of the overlying nerve fibers [8]. 4) With reference to the most discriminating SD-OCT areas, the AUC analyses showed that the cutoff values of ≥ 110 μm for the average thickness, or ≥ 150 μm for the inferior quadrant thickness were independently effective in their ability to discriminate ODE from PODE, with accuracy ≥0.70. The corresponding PPV was 82% for the average and 83% for the inferior quadrant, and it reached values even higher for participants aged ≥ 36 years. Since we investigated the usefulness of the OCT as first line examination, the cut offs were chosen in order to maximize the sensitivity. This was thought in order to limit the possible lack of ODE cases which, as such, need a prompt management.

The principal limitations of our study are the followings. 1) We did not formally investigate other sensible OCT parameters (e.g. the ONH volume, the presence of peripapillary wrinkles, retinal folds, subretinal peripapillary fluid accumulation, Bruch’s membrane opening size). These can improve the diagnostic aptitude of the sole peripapillary RNFL thickness measurement [15]. 2) The cutoffs in this study pertained to a specific SD-OCT instrument. Thus, our data are applicable only to this instrument. It would be interesting to test the reliability of these cutoffs by using other commercially available SD-OCT instruments. 3) Furthermore, PPV values can be influenced by the prevalence of the disease (ODE in this case). It will be valuable to validate the performance of these chosen cutoffs of RNFL measurements in different clinical settings and in participants of different ethnicity, as all patients in our study were Caucasians and it is well known that RNFL thickness varies by race/ethnicity [25,26]. In conclusion, although the correct differential diagnosis between ODE and PODE still relies on a careful medical history taking and other ancillary testing, the OCT RNFL analysis may help the initial selection of patients presenting with elevated optic disc to direct towards a prompt and extensive management.

## Supporting information

S1 Table Anonymous dataset: Group 0 corresponds to controls, group 1 to ODE and group 2 to PODE. (XLS)
